# Oxytocin has ‘tend-and-defend’ functionality in group conflict across social vertebrates

**DOI:** 10.1098/rstb.2021.0137

**Published:** 2022-05-23

**Authors:** Zegni Triki, Katie Daughters, Carsten K. W. De Dreu

**Affiliations:** ^1^ Department of Zoology, Stockholm University, Stockholm, Sweden; ^2^ Department of Psychology, Essex University, Colchester, UK; ^3^ Institute of Psychology, Leiden University, Leiden, The Netherlands; ^4^ Center for Research in Experimental Economics and Political Decision Making, University of Amsterdam, Amsterdam, The Netherlands

**Keywords:** parochial altruism, in-group, out-group, neuromodulation, decision-making, vertebrates

## Abstract

Across vertebrate species, intergroup conflict confronts individuals with a tension between group interests best served by participation in conflict and personal interest best served by not participating. Here, we identify the neurohormone oxytocin as pivotal to the neurobiological regulation of this tension in distinctly different group-living vertebrates, including fishes, birds, rodents, non-human primates and humans. In the context of intergroup conflict, a review of emerging work on pro-sociality suggests that oxytocin and its fish and birds homologues, isotocin and mesotocin, respectively, can elicit participation in group conflict and aggression. This is because it amplifies (i) concern for the interests of genetically related or culturally similar ‘in-group’ others and (ii) willingness to defend against outside intruders and enemy conspecifics. Across a range of social vertebrates, oxytocin can induce aggressive behaviour to ‘tend-and-defend’ the in-group during intergroup contests.

This article is part of the theme issue ‘Intergroup conflict across taxa’.

## 1. Introduction

Interactions between groups of conspecifics can be cooperative and benign but also hostile, for example, when groups compete for (access to) food, mating opportunities and territory [1,2]. Moreover, across species and all else equal, groups are more likely to be victorious when their members contribute to the collective aggression of rivalling other groups and prevent defeat when they contribute to the collective defence against enemy attacks [3,4]; and yet, joining conflict requires investing personal resources and increases the risk of injury. Participating in out-group aggression and in-group defence thus requires individuals to solve a tension between personal interests on the one hand and group interests on the other [4–7].

The tension between personal interests, served by withholding participation in conflict, and group interests served by pro-actively contributing, is seen in several species across taxa [1,7]. Perhaps there are evolutionary preserved biological mechanisms that regulate individual participation in intergroup conflict. Here, we examine this possibility at the neurobiological level by focusing on the role of oxytocin (and its homologues isotocin and mesotocin [8,9]) in regulating key parameters underlying conflict participation. We uncover a remarkable cross-species commonality in how isotocin in social fishes, mesotocin in gregarious birds, and oxytocin in group-living mammals biologically prepares for a ‘tend-and-defend’ response during intergroup conflict and not, or less so, for the aggressive subordination and exploitation of rivalling groups of conspecifics.

## 2. Evolution and neurobiology of oxytocin

**Figure 1.**
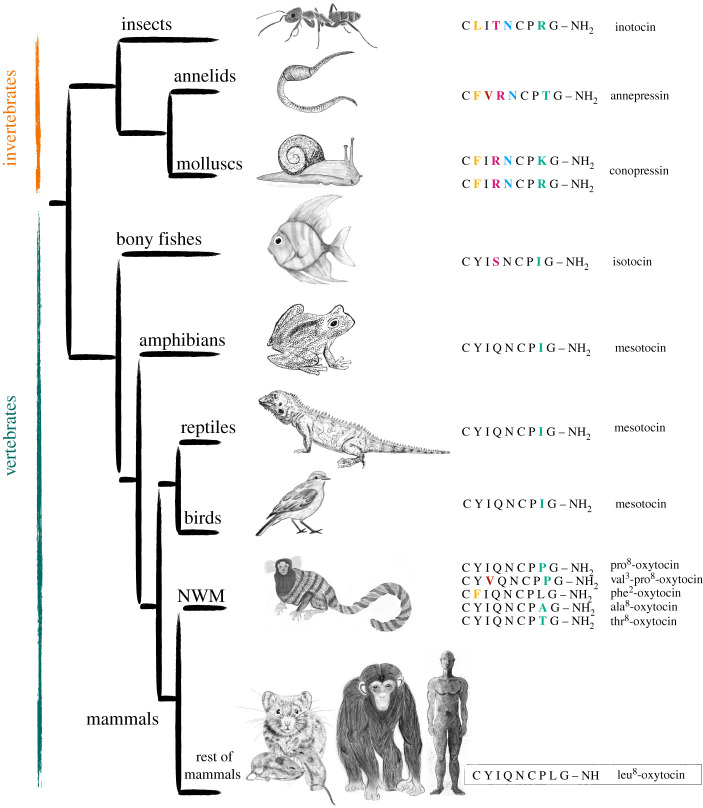
Oxytocin amino acids sequences across taxa/species. A simplified phylogenetic tree shows oxytocin sequence in different species and taxa with the common ancestors of oxytocin and vasopressin in invertebrates. To differentiate oxytocins in mammals, the variant amino acid and its position are indicated as a prefix. The leu^8^-oxytocin is taken here as a reference to see which amino acid(s) differ from this structure, where non-matching amino acids are colour coded. NWM refers to new world monkeys (e.g. marmosets, spider monkeys, capuchin monkeys, etc.). Nonapeptides sequences are from [8,12]. Illustrations by Z. Triki.

Oxytocin is a nine-amino acid peptide (i.e. nonapeptide) synthesised primarily in the brain. It can act centrally as a neuromodulator and/or peripherally as a hormone [9]. Across taxa and species, the mammalian oxytocin has several homologues, such as ‘isotocin’ in bony fishes [10], ‘mesotocin’ in nonmammalian tetrapods (lungfish, amphibians, reptiles and birds) [11], and up to five structural variants of oxytocin recently sequenced in new world monkeys [12] (figure 1).

Oxytocin can thus be viewed as an ancient peptide widely preserved across taxa. It shares common ancestors with another nonapeptide, ‘vasopressin’, that can be traced back all the way to snails and insects (figure 1). However, not all insects have this nonapeptide gene ancestor, such as silkworms, fruit flies, mosquitos, spiders and honeybees, suggesting a potential loss of such genes [13]. The vertebrates witnessed the emergence of oxytocin and its sister nonapeptide vasopressin (and their homologues) about 500 million years ago through gene duplication of a common ancestral gene, presumably in jawless fishes [8]. Since then, the oxytocin structure has remained highly preserved, from bony fishes to mammals, where the structural differences between oxytocin and its homologues and the recently discovered mammalian variants occur in one or two amino acids (figure 1). These differences notwithstanding, oxytocin variants and homologues thus share important structural and functional elements. For readability, we use here the nomenclature ‘oxytocin’ for all mammalian oxytocins, isotocin and mesotocin [14].

In all vertebrates, oxytocin is synthesised mainly in the magnocellular and parvocellular hypothalamic neurons. From here, oxytocin can be released centrally or relayed to the posterior pituitary gland, where oxytocin is released into the bloodstream and eventually cleared out in other fluids such as saliva and urine (box 1). In teleost and amphibians, the hypothalamic parvocellular and magnocellular neurons are located in the preoptic area and anterior hypothalamus. In other vertebrates, such as reptiles, birds and mammals, rather two separate nuclei, the paraventricular and supraoptic nuclei, harbour the oxytocin neurons (for further details, see [50]).

Box 1. **Measuring and manipulating oxytocin in social vertebrates.**

The box summarises the most common techniques used in biology to manipulate and measure oxytocin levels across taxa. To study causal effects, several methods exist to manipulate oxytocin (left panel). Non-invasive intra­nasal administration of oxytocin or an antagonist is commonly used in (non-)human primates. Invasive techniques include injections and intracerebro­ventricular infusions and are more commonly used in small mammals, fishes, and birds. Finally, causality is studied by comparing models with versus without intact oxytocin circuitry (i.e. knockdown (out) models). To examine correlations between naturally occurring oxytocin release and behaviour, central oxytocin is obtained from cerebrospinal fluid or directly from the brain, and peripheral oxytocin can be obtained from blood plasma, saliva, or urine (top right panel). Various assaying techniques exist to detect the presence of the nonapeptide in the sample (bottom right panel). For further details on the different techniques' accuracy and validity and the extent to which endogenous/exogenous oxytocin levels can be an informative tool in behavioural studies, please see [15–22].

**Table:** 

manipulating (oxytocin agonist/antagonist)				measuring			
***non-invasive***	***nasal spray***	***nebuliser***	***nasal drops***	***central***	***cerebrospinal fluid***	***microdialysis***	***brain harvesting***
	primates [23]	primates [24]	rodents [25]		primates [26]	rodents [25]	rodents [27]
			birds [28]		rodents [29]		birds [30]
							fishes [31]
**invasive**	**intravenous/intra-muscular/intraperitoneal**	**intracerebroventricular infusion**		**peripheral**	**blood plasma**	**saliva**	**urine**
	primates [32]	primates [33]			primates [26]	primates [23]	primates [34]
	rodents [25]	rodents [35]			carnivores [36]	birds [37]	carnivores [38]
	birds [30]	birds [39]			rodents [25]		
	fishes [40]	fishes [41]			birds [42]		
					fishes [43]		
**genetic**	**knockout models**	**knock-down models**	**behavioural phenotypes (selection experiments)**	**assay**			
	rodents [44]	rodents [27]	rodents [45]	enzyme-linked immunosorbent assay (ELISA) [23]			
		birds [46]		radioimmunoassay (RIA) [19]			
				liquid chromatography-mass spectrometry (LC-MS) [47]			
				immunohistochemistry (IHC) [48]			
				mRNA quantification [49]

Upon its release from neuronal soma, axons and dendrites, oxytocin exerts widespread effects in the brain via an oxytocin-specific G protein-coupled receptor [9]. Oxytocin binding on this receptor activates a set of signalling cascades that can quickly modulate the evolutionary ancient and structurally and functionally preserved social decision-making network in the vertebrate brain [51,52]. This network includes various brain nuclei known for their crucial roles in regulating social recognition, affiliation and parental behaviour, responses to social stressors and aggression [2,52–54].

## 3. Oxytocin and participation in group conflict

There is growing evidence that in a range of species, oxytocin plays a significant role in forming and solidifying social structures (e.g. [15, 55]. In particular, affiliation among conspecifics is often associated with higher oxytocin levels. For example, studies that use oxytocin levels from blood plasma, urine or saliva as an informative tool on central oxytocin release, have recorded elevated oxytocin following affiliative touch [56–58] and cooperative exchange [59–61] in mammalian species such as (human) primates and dogs. Also, strongly bonded marmoset monkeys showed synchronized fluctuations of oxytocin over a six-week period [62] (also see [63]). Similar positive effects of affiliation on oxytocin levels are found in gregarious birds [64], lizards [65] and fishes [31]. Other work observed links between oxytocin levels in distinct brain regions on the one hand, and a range of social behaviours on the other, including suckling (in rats and sheep [66–68] and mating (e.g. in voles [69]).

**Figure 2.**
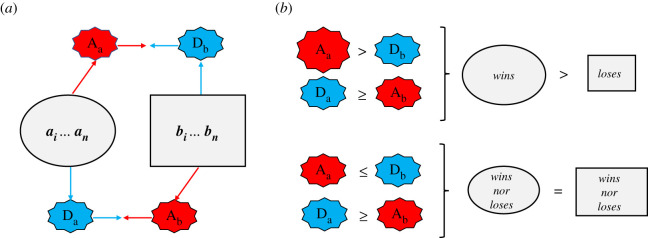
Intergroup conflict as a multilevel contest game of strategy. (*a*) Individuals nested in two groups (circle: *a_i_ … a_n_* and square: *b_i_ … b_n_*) can contribute personal resources (e.g. skills, time and energy) to their group's capacity for out-group attack A; red) and/or to protect against enemy attacks (in-group defence D; blue). Conflict participation is risky–the individual may get injured–and resources contributed are ‘wasted’. (*b*) Conflict participation increases the likelihood of victory with concomitant ‘spoils of war’ (a win/lose outcome; top panel), and of surviving out-group attacks (a stalemate outcome; bottom panel). Because (participating in) conflict is wasteful, even winning groups typically are less wealthy post-conflict.

At first blush, the mutually reinforcing relationship between affiliation and oxytocin may appear antagonistic to the possibility that oxytocin prepares individuals for participation in hostile group conflict with conspecifics. However, for group conflict to be won, or not lost, individuals within rivalling groups need to contribute to their group's fighting capacity at some personal cost (figure 2) (also see [2,5–7,70,71]). Making such costly contributions serves the group and can thus be seen as a form of pro-social behaviour towards one's in-group. Indeed, as we [2,72] and others (e.g. [1,5,7]) have argued and shown, in many group-living species an individual's conflict participation *p*_i_ is a function of concern for in-group (henceforth *α*_I_) and out-group interests (henceforth *α*_O_), expected out-group threat (henceforth *β*), and compliance with group norms for participation (*viz*. reputation concerns; henceforth *γ* (see also [73]. If we set each parameter to vary between −1 and 1 inclusive, participation likelihood increases when there is a positive concern for in-group interests (*α*_I_ > 0), negative concern for out-group interests (*α*_O_ < 0), perceived out-group threat (*β* > 0) or when the animal expects participation returns reputation benefits (*γ* > 0) [1,2,4,73]. This means that participation can be expected when and because oxytocin increases (i) in-group concern *α*_I_, and/or (ii) creates negative out-group concern (*α*_O_ < 0), and/or (iii) increases perceived out-group threat *β*, and/or (iv) increases expectation of reciprocity and reputation benefits from participating (*γ*). In the remainder of this section, we examine the evidence for the role of oxytocin on each of these parameters underlying participation in conflict (also see [68,74–77]) (box 2).

Box 2. **Inferring conflict participation parameters from vertebrate decision-making.**

*Concern for in-group (α_I_) and out-group (α_O_)* can be inferred from behavioural choices, neural activation in, e.g. mesolimbic reward circuitry and, in humans, self-reports. In humans, social concerns can be inferred from economic decision-making games such as the Dictator Game (DG), wherein participants donate *x* out of an endowment *e* to an anonymous recipient (with 0 ≤ *x* ≤ *e*). Higher donations to in-group rather than out-group members reflect stronger concern for in-group (*α*_I_) than out-group interests (*α*_O_) [78,79]. Variants of such games have been used to infer social preferences in non-human primates [80]. In nonmammalian vertebrates, such as social fishes, social preferences are inferred from time spent in proximity of a conspecific [40] or from costly helping of a conspecific [81]. To infer *expectations of reciprocity* (viz. *β*), studies with humans used trust games. Participants can transfer *x* out of an endowment *e* to a recipient (with 0 ≤ *x* ≤ *e*). The recipient then receives 3*x* and can return *y* to the participant (with 0 ≤ *y* ≤ 3*x*). Greater transfers reflect expectations of reciprocity (or ‘trust’), and greater back-transfers reflect a willingness to reciprocate (or ‘trustworthiness’) [79]. Vice versa, expectations of competition can be inferred from partner choice, with rejecting partners who did not cooperate on earlier occasions as a measure of negative expectations (in humans[79]; in birds [82]; in fishes [83]). Finally, *reputation concerns* have been inferred from third-party punishment games [80], where participants, after decision-making, express through punishment social disapproval of the others' (non-cooperative) behaviour and/or induce a norm for cooperation on future trials [84]. Punishment and behavioural adjustments to (threat of) punishment are seen across social vertebrates, including chimpanzees [85] and social fishes [86].

### (a) Parochial preferences (in-group interest *α*_I_ > out-group interest *α*_O_)

Studies with human participants revealed that concerns for genetically related or culturally similar conspecifics (in-group) are typically stronger than for unrelated and unfamiliar (out-group) conspecifics [78] (box 2). Oxytocin has a mechanistic role to play in such in-group biased preferences (i.e. *α*_I_ > *α*_O_) [87,88]. For instance, in-group participants in a foraging game helped each other more often compared to out-group participants, a behaviour that was mediated by endogenous oxytocin (i.e. measured in saliva) [59]. Similarly, Chinese males had a frontocentral positive activity of larger amplitude in response to the pain expressions of in-group (Asian targets) but not out-group members (Caucasian targets), especially following intranasal administration of oxytocin rather than placebo [89] (also [90]).

In humans, oxytocin seems to amplify *α*_I_ and neither increases nor decreases *α*_O_—oxytocin makes humans like their in-group more and does not condition (dis)liking out-groups. This was shown, for example, when human participants indicated their liking for individuals from their own nationality (i.e. Dutch citizens) and individuals from a more or less rivalling nationality (e.g. Germans). Compared to placebo-treated individuals, those given intranasal oxytocin expressed a greater liking for in-group members (an increase in *α*_I_) but did not increase or decrease their liking for out-group members (i.e. *α*_O_ was similar in oxytocin and placebo conditions) [87]. Recent work on wild chimpanzees suggests that these effects may generalize to other species, including voles [91,92], sheep [93] and chimpanzees [94]). In another series of experiments with human participants, individuals were organised in two groups of three and could contribute to club goods A and B out of a personal endowment. Whereas contributions to A and B equally benefitted the members of one's own group, contributions to B (but not A) also imposed a cost on the out-group members. Intranasal oxytocin (versus placebo) increased contributions to club good A, reflecting an increase in *α*_I_. However, oxytocin neither increased nor decreased contributions to club good B, suggesting oxytocin did not affect *α*_O_ [95,96] (also see [97,98]).

Although follow-up experiments in humans sometimes show that oxytocin can increase *α*_O_ (e.g. [99,100]), this effect is rarely as strong as the oxytocin-induced increase on *α*_I_. This mirrors findings with non-human vertebrates. For example, marmosets treated with marmoset-specific pro^8^-oxytocin reduced pro-sociality towards strangers compared to those treated with saline or consensus-mammalian leu^8^-oxytocin [101] (see also [102]). Chimpanzees had higher urinary oxytocin concentrations before and after hostile intergroup encounters, which predicted within-group affiliative behaviours [34]. Resident male mice exhibit higher attack bites against intruders of different strains (*viz.* out-group) than against intruders of their own strain. Yet compared to oxytocin receptor wild-type mice, oxytocin receptor-null residents exhibited greater aggression towards intruders of their own strain, suggesting that oxytocin modulates *α*_I_ more than *α*_O_ [44]. In a monogamous zebra finch, affiliation towards one's partner requires the activation of the oxytocin receptor [39], while oxytocin knockdown birds and those treated with an oxytocin antagonist experienced affiliation behaviour deficit [46,64] (for similar findings in pinyon jays, see [25]). Finally, work on the mutualistic cleaner fish and its various coral reef fish clients showed that cleaners injected with oxytocin break less often the already engaged cleaner-client social interaction to initiate a new interaction with a newly arrived client [103].

Together, there is growing evidence for the possibility that across social vertebrates, oxytocin appears to increase a positive concern for the interests of familiar conspecifics more than for the interests of genetically or culturally unfamiliar, out-group conspecifics: *α*_I_ > *α*_O_. At least in humans, this parochial preference is also reflected in in-group-biased expectations of reciprocity derived from trust games (box 1; [74,95]). In short, when individuals with elevated levels of oxytocin participate in conflict this is more likely owing to an increase in *α*_I_ than because of a decrease in *α*_O_.

### (b) Responding to out-group threat (*β*)

Nursing rats protect their offspring against intruders by aggressing them with fast attacks directed towards the intruder's neck or back region, lateral threats to force the intruder aside, and standing in an upright posture in front of the intruder, sometimes using the front legs to hold the intruder down [104]. Such ‘maternal defence’ rests on oxytocin, where oxytocin knockout rats and those treated with oxytocin antagonists abstain from aggressing intruders [54,104,105].

A suite of follow-up studies shows oxytocin-mediated aggression towards threatening outsiders is not confined to (female) rodents. For example, when groups of wild meerkats were given intravenous oxytocin (or placebo), individuals spent over twice as much time ‘on guard’; a personally costly behaviour that helps to protect the group against an outside threat from predators and hostile conspecifics [106] (see [102] for similar results in marmoset monkeys). Likewise, estrildid finches that form year-round male-female pairs aggressively defend their territories from intruders. Yet, such aggressive defence is significantly reduced following the blockade of oxytocin receptors in the avian brain [30]). Also, in social fishes such as cichlids and sticklebacks, the presence of an intruder incites higher oxytocin neuronal activity [48] (also see [107]), and higher brain oxytocin levels associate with an aggressive defence of nest and territory [31].

Experiments with human participants confirmed that oxytocin could elicit defensive aggression and suggest that such aggression is closely tied to rivalling out-group threats. For example, several studies showed that oxytocin increases competition against out-group members if, and only if, out-group hostility would hurt the individual and/or its in-group members [95,108] (also see [76]). Other studies using different experimental tasks produced similar results. For example, individuals given oxytocin more quickly (and less accurately) aggressed ethnically different rather than ethnically similar intruders [109].

Taken together, there is converging evidence across social vertebrates that oxytocin upregulates attention and aggressive responses towards predators and rivalling conspecifics. In addition to parochial preferences (§3a), individuals with elevated levels of oxytocin may increase their conflict participation because of enhanced perception of out-group threat and increased readiness to protect and defend genetically related and culturally familiar conspecifics [110].

### (c) Reputation and group norms for participation (*γ*)

Individuals within groups adapt behaviour to other group members' choices, including those of ‘first-movers’ and group leaders [111,112]. Such behavioural alignment or ‘compliance’ enables the individual to benefit from the protection offered by the group and, in addition, facilitates the coordination of collective action towards some group goal [79,113]. Behavioural alignment thus is functional towards both individual and group survival and prosperity both in general and in the context of intergroup conflict. Furthermore, groups are more likely to win intergroup contests when individual contributions are well-coordinated and aligned with leader initiatives [88,98,114] (also see [73]).

There is some evidence that oxytocin facilitates behavioural alignment and compliance with group norms. Humans, for instance, change their private views in the direction of their group members' opinions more when given oxytocin rather than placebo [115–118]. Likewise, oxytocin mediates interpersonal synchronization at both the neural and behavioural levels in humans [119–123], marmoset monkeys [62], dogs [124] and social fishes [125]. In one study with humans, individuals within groups aligned their contributions to group conflict better when given oxytocin than placebo. As a result, their groups won greater ‘spoils of war’ [98]. Oxytocin may, therefore, prepare the individual for conflict participation because it increases sensitivity to and compliance with leader initiatives and group norms for participation.

Because compliance can have adaptive functionality to the group, individuals are willing to enforce compliance in other group members [79,84]. For example, humans punish those who fail to contribute to group conflict, and such (threat of) punishment increases subsequent conflict participation [114]. At least in humans, there is some evidence that oxytocin prepares the individual for such norm enforcement. For instance, in one study, participants as neutral third parties punished group members who had exploited another person's trust more when given oxytocin rather than placebo [126] (also see [127–130]). In short, oxytocin facilitates interpersonal synchronization and alignment across various social vertebrates at the neural, physiological and behavioural levels. Possibly, and especially when collaborations require strong synchrony in space and time [131], individuals with elevated oxytocin may participate in group conflict because of amplified *γ*—the readiness to align with and follow other group members' initiatives.

## 4. Conclusion

**Figure 3.**
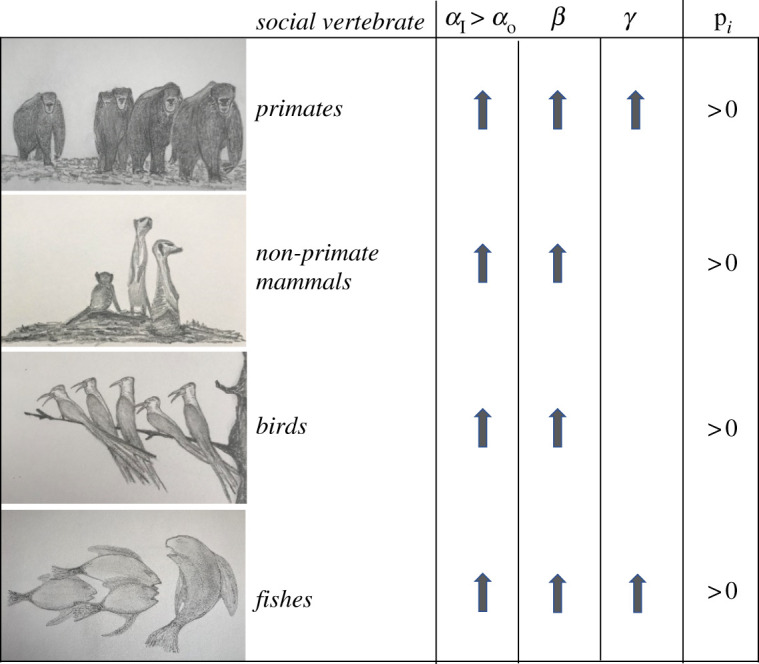
Oxytocin and conflict participation parameters across social vertebrates. Oxytocin creates parochial preferences (*α*_I_ > *α*_o_) because it upregulates *α*_I_ (concern for genetically and culturally related conspecifics) and less *α*_O_ (concern for genetically and culturally unrelated conspecifics). Oxytocin also upregulates *β* (the willingness to defend aggressively against intruders and groups of rivalling conspecifics). At least in primates, oxytocin increases *γ* (behavioural alignment with group norms for participation). Arrows indicate the direction of an effect of, or association with oxytocin. Empty cells indicate no or too little evidence is available. Illustrations by C. De Dreu.

Our review reveals converging evidence for the possibility that oxytocin has a ‘tend-and-defend’ functionality that prepares for active conflict participation through an increase in parochial in-group preferences (*α*_I_) and perceived threat from out-groups (*β*). We observed little to no evidence that oxytocin modulates (negative) concern for out-groups (*α*_O_) and concomitant aggression aimed at exploiting and sub-ordinating outsiders (figure 3).

Our conclusion comes with some limitations. First, we allowed for some degrees of freedom in interpreting animal behaviour as reflective of social preferences (*α*), threat-responding (*β*) and norm compliance (*γ*). Such ‘heuristic’ treatment ignores that both animal behaviour and hormones are often equifinal—different behaviours or hormones serving the same function—and multi-final—the same behaviour or hormone serving several functions [132–134]. Future experiments could try to isolate these parameters further and, in addition, examine possible interactions (e.g. social preferences upregulated threat-responding). Second, not all parameters in the conflict participation function have been covered across social vertebrates, and there are a range of context-dependencies that can complicate straightforward predictions. Conclusive evidence for oxytocin-induced reputation concerns and compliance with group norms, for example, appears limited to humans (figure 3). Third, our analysis collapsed across various measurements and manipulations of oxytocin, and some evidence is strictly correlational. For example, research with humans mostly relied on upregulating oxytocin and has not examined how oxytocin antagonists reduce conflict participation. Also, research often either considered only females or males, while some effects might be sex-specific.

The converging evidence for ‘tend-and-defend’ functionality across social vertebrates should not be taken as if oxytocin is required for participation in group conflict to emerge. Some highly social species such as bees engage in lethal intergroup conflict [135] yet lack oxytocin homologues. Whereas social vertebrates may have co-opted the oxytocinergic circuitry to support a ‘tend-and-defend’ response during the intergroup conflict, other species may rely on different neuroendocrine systems to produce strategic engagement in intergroup conflict. In addition, in social vertebrates, other neurohormonal mechanisms may contribute to conflict participation. For example, oxytocin and vasopressin co-evolved, where vasopressin differs in two amino acids compared to oxytocin [14]. Yet, like oxytocin, vasopressin regulates affiliative behaviour and context-dependent aggressive behaviour (e.g. competition, territory defence) [40,136]. Furthermore, the sex steroid testosterone mediates aggressive behaviour, which can influence group conflict outcomes [137], and the stress hormone cortisol mediates the natural ‘fight-or-flight’ response to threatening conspecifics [34]. Future work into the neurohormonal underpinnings of conflict participation is needed, particularly in how distinctly different neurotransmitters and hormones interact in producing prosocial behaviour towards genetically related and culturally similar conspecifics and aggression towards more or less rivalling out-groups.

## Data Availability

This article has no additional data.
